# Digital Approaches for Myositis

**DOI:** 10.1007/s11926-023-01119-4

**Published:** 2023-11-14

**Authors:** Johannes Knitza, Sebastian Kuhn, Latika Gupta

**Affiliations:** 1 Department of Internal Medicine 3, Rheumatology and Immunology Friedrich, Alexander University Erlangen-Nürnberg and Universitätsklinikum Erlangen, Erlangen, Germany; 2 AGEIS, Université Grenoble Alpes, Grenoble, France; 3 Institute of Digital Medicine, University Hospital of Giessen and Marburg, Philipps-University Marburg, Marburg, Germany; 4 Department of Rheumatology, Royal Wolverhampton Hospitals NHS Trust, Wolverhampton, UK; 5 City Hospital, Sandwell and West Birmingham Hospitals NHS Trust, Birmingham, UK; 6 Division of Musculoskeletal and Dermatological Sciences, Centre for Musculoskeletal Research, School of Biological Sciences, The University of Manchester, Manchester, UK

**Keywords:** Myositis, Telehealth, eHealth, Wearables, Digital

## Abstract

**Purpose of Review:**

This article serves as a comprehensive review, focusing on digital approaches utilized in the diagnosis, monitoring, and treatment of patients with idiopathic inflammatory myopathies (IIM). The authors critically assess the literature published in the last three years, evaluating the advancements and progress achieved in this specific domain.

**Recent Findings:**

Remarkable strides have been made in the realm of digital diagnostic support, particularly in image analysis and clinical prediction models, showing promise in aiding the diagnosis of IIM. The field of remote patient monitoring has also witnessed significant advancements, revolutionizing the care process by offering more convenient, data-driven, and continuous monitoring for IIM patients. Various digital tools, such as wearables, video- and voice consultations, and electronic patient-reported outcomes, have been extensively explored and implemented to enhance patient care. Survey studies consistently reveal a high acceptance of telehealth services among patients. Additionally, internet-based studies have facilitated the efficient and rapid recruitment of IIM patients for research purposes. Moreover, the integration of sensors and exoskeletons has shown great potential in significantly improving the functionality and quality of life for individuals with muscle weakness caused by IIM.

**Summary:**

The integration of digital health solutions in the care of IIM patients is steadily gaining attention and exploration. Although the existing evidence is limited, it does indicate that patients can be adequately and safely supported through digital means throughout their entire healthcare journey. The growing interest in digital health technologies holds the promise of improving the overall management and outcomes for individuals with idiopathic inflammatory myopathies.

## Introduction

Idiopathic inflammatory myopathies (IIM) encompass a group of chronic and rare autoimmune disorders primarily affecting the proximal muscles. The complexity, nonspecific symptoms, and rarity of these diseases contribute to a significant diagnostic delay [1]. Disease activity tends to fluctuate, with intermittent flares between medical visits, necessitating timely adjustments to immunosuppressive treatments. Current monitoring methods relying on face-to-face (F2F) visits pose significant burdens on patients with limited mobility and only provide a snapshot for healthcare professionals (HCPs). Access to specialized centres for IIM patients is limited and often challenging for patients to reach [2]. Treatment approaches for IIM patients remain suboptimal, often involving a trial-and-error process, and the provision of prescribed physiotherapy is lacking [1].

Technological advancements continue to revolutionize the healthcare landscape, offering substantial potential to enhance IIM healthcare significantly. Embracing a digitally supported patient pathway holds the promise of providing more patient-centric, convenient, cost-effective, continuous, and data-driven care. The COVID-19 pandemic has expedited the adoption of telehealth solutions, leading to increased acceptance among patients and rheumatologists [3, 4]. The benefits of telemedicine have been acknowledged by EULAR, as evidenced by their publication of points-to-consider for remote care in 2022 [5••]. These developments underscore the transformative role that digital health solutions can play in optimising the management and outcomes for individuals with inflammatory myopathies [6].

By critically evaluating the recent literature, we aim to provide a comprehensive understanding of the current state of digital health solutions for IIM and identify potential avenues for future research and implementation in clinical practice.

## Diagnosis

Digital health tools, particularly patient-facing diagnostic decision support systems (DDSS), have the potential to expedite the diagnosis of IIM patients. Rheumatic patients are increasingly utilizing such tools [4, 7], and they have been subject to investigation in a large multicentre trial for individuals with suspected rheumatic diseases [7–9]. The key advantage of these apps, also known as symptom checkers, lies in their accessibility and ability to provide a rapid initial assessment.

While patients have shown favourable perceptions towards DDSS, their diagnostic accuracy remains relatively low. This could be attributed to the limited information currently incorporated into these tools, primarily reliant on the presence or absence of symptoms. Enhanced access to a broader range of information is likely to improve diagnostic accuracy, as demonstrated in other studies, such as asynchronous telehealth assessments in patients with suspected axial spondyloarthritis [10]. Expanding the depth and breadth of information available to these digital tools could potentially enhance their efficacy in aiding the diagnosis of inflammatory myopathies and other rheumatic conditions. Determination of autoantibodies remains a diagnostic gold standard to differentiate the heterogenous forms of IIM. A noteworthy study demonstrated that patients with IIM can effectively collect capillary blood samples at home, and the analysis of these samples proves to be as accurate as venous blood collected in a hospital setting [11]. Patients showed great enthusiasm in avoiding unnecessary appointments solely for blood collection purposes, particularly when utilizing new upper-arm devices that were found to be almost painless [12]. At-home diagnostic assessment combining eHealth and patient self-sampling could significantly improve access to care and accelerate diagnosis.

Other digital medical devices enable earlier detection of relevant complications by augmenting physicians’ ability in the diagnostic process. Xue et al. built a machine learning algorithm, superior to a conventional regression model to predict the risk of antiMDA5 antibody in juvenile dermatomyositis children [13]; a fatal manifestation of IIMs is interstitial lung disease (ILD). Early identification, monitoring of the lung areas involved and correct classification of ILD pattern is crucial. Artificial intelligence is especially implemented in medical image analysis. A systematic review pointed out diagnostic accuracies of 78%–91%, with two studies demonstrating near-expert performance. However, most studies (78.9%; 15/19) had a high risk of bias.

## Monitoring

A recent international survey among rheumatologists showed that the majority of participants felt that telemedicine is inadequate for the monitoring of IIM flares, compared to flares of arthritis [14]. Disease complexity, lack of financial reimbursement and lack of physical examination were main identified barriers, in line with previous work [15, 16]. Nevertheless, during the COVID pandemic remote monitoring for IIM patients was implemented at some centres. Cavagna et al. presented results of their telehealth implementation during the pandemic for patients with connective tissue disease, including IIMs [17•]. Confirming previous results [18], patients’ attitude towards telehealth correlated with the distance from the hospital. Patients with a higher education were more likely to prefer telemedicine over in-person visits. Lack of telemedicine skills was a main barrier, highlighting eHealth literacy [19] as major factor for successful implementation of telehealth. Naveen et al. also offered IIM patients’ telehealth during the pandemic [20•]. Voice consultations were the preferred medium and nearly one third of the patients consulted in emergency cases. In this study, the authors did not observe differences between telehealth users and non-users regarding their demographic and socioeconomic profile.

Most of the recent digital health publications regarding IIMs concern disease monitoring. The clinical gold-standard are collected during F2F visits, enabling only a limited analysis of disease activity. Digital health, including wearables, videoconsultations, mobile apps and ePRO (electronic patient-reported outcomes) enable a more continuous monitoring of disease activity, complementing F2F visits. The current gold-standard to assess muscle strength included as a core set measure (CSM) in the official ACR/EULAR total improvement score (TIS) [21] is manual muscle testing (MMT-8). The test remains the gold-standard despite being very subjective, subject to large interobserver variability and exhibiting ceiling effects [22]. Focusing on muscle endurance instead of direct muscle strength testing tries to overcome these barriers. The clinical gold standards to measure muscle endurance are the functional index (FI) [23] and 6-min walking distance (6MWD). The latest and third version of the FI includes three muscle groups and takes less than 10 min: shoulder flexion, neck flexion and hip flexion. One disadvantage is that patients are required to complete the repetitions with a specific weight and at a set rhythm. Additionally, the FI and 6MWD are performed in the hospital under HCP supervision. Patient-performed tasks and device-based measurements enable remote monitoring of disease activity.

Validation of remote assessments with IMACS core set outcomes is the unmet need of the hour. Ravichandran N et al. recently evaluated two novel patient–performed tests, namely ten times arm lift (AL) test and 2-min walk distance (2MWD) in IIM patients in a telehealth myositis patient cohort [24•]. AL correlated well with all CSMs except CK levels and MDI. 2MWD values were highly variable and showed no correlation with CSMs.

Wearables have the advantage of continuous and passive data collection. Rockette-Wagner et al. validated a waist-worn sensor (ActiGraph GT3X-BT) [25]. Measurements showed good TIS responsiveness. The same group compared the accuracy of the expensive ActiGraph® with the more affordable Fitbit® devices and could demonstrate good agreement and strong correlation (ICC 0.96) for step count [26••]. Milazzo et al. published a pilot study with wearable sensors to objectively quantify muscle strength of the upper limb muscles in patients with muscle dystrophies [27]. Whereas the current system is still quite bulky, a point-of-care solution for at home monitoring seems feasible. Using data collected passively from smartwatches and/or smartphones, a self-administered 6-min walk distance is very convenient for patients [28].

Established questionnaires can easily be distributed electronically. Implementation of a smartphone app and a weekly questionnaire unveiled the high frequency of disease flares in IIM patients [29]. The most commonly used questionnaires in IIM are Health Assessment Questionnaire (HAQ-DI) and SF-36 physical function-10 (PF10). Recently Yoshida et al. established the dispersion of patient reported physical function in IIM [30], and a global patient led e-survey identified the spectrum of pain [31] experienced by patients using validated PROs.

## Treatment

Effective remote monitoring could provide a more solid and detailed rationale for treatment decisions. Despite the recent advances in drug treatment of IIMs [32, 33], some patients experience are very refractory and can no longer manage their daily lives independently. Wearables such as a cyborg Hybrid Assistive Limb investigated by Nakajima et al. [34] could help patients to cope better with everyday life. In a multicentre randomized controlled trial in patients with neuromuscular diseases patients using the wearable device experienced significant improvements regarding 2MWD and MMT scores. For the Mysosuit, a robotic exoskeleton-assisted wearable, first promising results have also been reported in a pilot study with lung transplantation candidates [35].

Internet-based research is invaluable for rare diseases such as IIM to accelerate research [36]. A recent large international survey study in patients with antisynthetase syndrome enabled a detailed picture of healthcare utilization and unmet patient needs [1]. The study unveiled a general lack of prescribed physiotherapy and heterogenous treatment patterns. Patients were most interested in additional disease related information and preferred online information compared to local community workshops. Joshi et al. systematically identified and evaluated the quality of YouTube videos for patient and physician education on inflammatory myositis [37]. A total of 900 videos were identified and 74% of the videos were classified as useful. The authors conclude that HCPs should recommend and provide their patients with high-quality information material. A recent app-based pilot study prompting patients’ evidence-based information suggested an improvement of quality of life for arthritis patients [38] and offer for IIM patients would likely just be as useful.

## Research

Social media driven internet-based research allows greater outreach and data collection for rare rheumatic diseases such as IIM. This is best exemplified by the COVAD group, a focussed patient reported e-survey floated by a collaborative group of experts covering 106 countries and 164 centres [39]. This group identified global disparities in treatment of IIM, outlining advocacy for rare rheumatic diseases as an important agenda for collaborative study groups with worldwide representation [40]. Hannah et al. demonstrated that based on routinely collected clinical data, namely ICD codes, IIM patients can be accurately identified to enable more cost-effective research [41]. A combination of ICD codes improved diagnostic accuracy, paving the way to optimise the use of registry-based datasets for rare disease research.

## Conclusion

Given the limited availability of specialized centres and the severe functional limitations caused by IIM, telehealth emerges as an ideal solution for improving patient care. This review emphasizes the substantial progress achieved in recent years, although large and robust studies in this area are still relatively scarce. The majority of identified studies have focused on remote patient monitoring, indicating a growing interest in this approach. Both patients and healthcare professionals express a strong desire for a more detailed and continuous overview of disease activity between face-to-face appointments. The integration of ePROs, self-collection of blood samples, and wearables empowers patients to take on a more active role in managing their condition.

Notably, the implementation of telehealth could also pave the way for a real-world data registry, facilitating patient recruitment, and even enabling decentralized clinical trials. These advancements have the potential to significantly enhance the overall management and research related to idiopathic inflammatory myopathies, offering greater convenience and improved outcomes for patients.

## Data Availability

The data that support the findings of this study are available from the corresponding author, Johannes Knitza, upon reasonable request.

## References

[1] Weiss M, Holzer MT, Muehlensiepen F, et al. Healthcare utilization and unmet needs of patients with antisynthetase syndrome: an international patient survey. Rheumatol Int. 2023; 10.1007/s00296-023-05372-9 10.1007/s00296-023-05372-9 PMC10435645 37452880

[2] Masanneck L, Räuber S, Schroeter CB (2023). Driving time-based identification of gaps in specialised care coverage: an example of neuroinflammatory diseases in Germany. Digit Health.

[3] Saud A, Naveen R, Aggarwal R, Gupta L (2021). COVID-19 and Myositis: What We Know So Far. Curr Rheumatol Rep.

[4] Kernder A, Morf H, Klemm P (2021). Digital rheumatology in the era of COVID-19: results of a national patient and physician survey. RMD Open.

[5] de Thurah A, Bosch P, Marques A (2022). 2022 EULAR points to consider for remote care in rheumatic and musculoskeletal diseases. Ann Rheum Dis annrheumdis.

[6] Gupta L, Najm A, Kabir K, De Cock D (2023). Digital health in musculoskeletal care: where are we heading?. BMC Musculoskelet Disord.

[7] Knitza J, Muehlensiepen F, Ignatyev Y, et al. Patient’s perception of digital symptom assessment technologies in rheumatology: results from a multicentre study. Frontiers. Public Health. 2022;10 10.3389/fpubh.2022.844669 PMC8902046 35273944

[8] Gräf M, Knitza J, Leipe J, et al. Comparison of physician and artificial intelligence-based symptom checker diagnostic accuracy. Rheumatol Int. 2022; 10.1007/s00296-022-05202-4 10.1007/s00296-022-05202-4 PMC9548469 36087130

[9] Knitza J, Mohn J, Bergmann C (2021). Accuracy, patient-perceived usability, and acceptance of two symptom checkers (Ada and Rheport) in rheumatology: interim results from a randomized controlled crossover trial. Arthritis Res Ther.

[10] Hannah L, von Sophie R, Gabriella RM, et al. Stepwise asynchronous telehealth assessment of patients with suspected axial spondyloarthritis: results from a pilot study. Rheumatol Int. 2023; 10.1007/s00296-023-05360-z 10.1007/s00296-023-05360-z PMC10766678 37316631

[11] Zarbl J, Eimer E, Gigg C (2022). Remote self-collection of capillary blood using upper arm devices for autoantibody analysis in patients with immune-mediated inflammatory rheumatic diseases. RMD Open.

[12] Muehlensiepen F, May S, Zarbl J (2022). At-home blood self-sampling in rheumatology: a qualitative study with patients and health care professionals. BMC Health Serv Res.

[13] Xue Y, Zhang J, Li C (2022). Machine learning for screening and predicting the risk of anti-MDA5 antibody in juvenile dermatomyositis children. Front Immunol.

[14] Chock EY, Putman M, Conway R (2022). Experience with telemedicine among rheumatology clinicians during the COVID-19 pandemic: an international survey. Rheumatol Adv Pract.

[15] Muehlensiepen F, Knitza J, Marquardt W (2021). Acceptance of telerheumatology by rheumatologists and general practitioners in Germany: nationwide cross-sectional survey study. J Med Internet Res.

[16] Muehlensiepen F, Petit P, Knitza J (2023). Factors associated with telemedicine use among patients with rheumatic and musculoskeletal disease: secondary analysis of data from a German nationwide survey. J Med Internet Res.

[17] Cavagna L, Zanframundo G, Codullo V (2021). Telemedicine in rheumatology: a reliable approach beyond the pandemic. Rheumatology (Oxford).

[18] Vossen D, Knitza J, Klemm P, et al. Acceptance of video consultation among patients with inflammatory rheumatic diseases depends on gender and location-results of an online survey among patients and physicians. Z Rheumatol. 2021; 10.1007/s00393-021-01052-w 10.1007/s00393-021-01052-w PMC8391858 34448915

[19] Knitza J, Simon D, Lambrecht A (2020). Mobile health usage, preferences, barriers, and ehealth literacy in rheumatology: patient survey study. JMIR Mhealth Uhealth.

[20] Naveen R, Sundaram TG, Agarwal V, Gupta L (2021). Teleconsultation experience with the idiopathic inflammatory myopathies: a prospective observational cohort study during the COVID-19 pandemic. Rheumatol Int.

[21] Aggarwal R, Rider LG, Ruperto N (2017). 2016 American College of Rheumatology/European League Against Rheumatism criteria for minimal, moderate, and major clinical response in adult dermatomyositis and polymyositis: an International Myositis Assessment and Clinical Studies Group/Paediatric Rheumatology International Trials Organisation Collaborative Initiative. Ann Rheum Dis.

[22] Gregory WJ, Saygin D (2022). Assessment of physical activity and muscle function in adult inflammatory myopathies. Curr Rheumatol Rep.

[23] Ernste FC, Chong C, Crowson CS (2021). Functional index-3: a valid and reliable functional outcome assessment measure in patients with dermatomyositis and polymyositis. J Rheumatol.

[24] Naveen R, Thakare DR, Agarwal V (2022). Validation of two simple patient-centered outcome measures for virtual monitoring of patients with idiopathic inflammatory myositis. Clin Rheumatol.

[25] Rockette-Wagner B, Saygin D, Moghadam-Kia S (2021). Reliability, validity and responsiveness of physical activity monitors in patients with inflammatory myopathy. Rheumatology (Oxford).

[26] •• Saygin D, Rockette-Wagner B, Oddis C, et al. Consumer-based activity trackers in evaluation of physical activity in myositis patients. Rheumatology (Oxford). 2021:keab700. **Validation study highlighting the value of activity trackers.**10.1093/rheumatology/keab700 10.1093/rheumatology/keab700 34528065

[27] Milazzo M, Spezzaneve A, Astrea G (2021). AUTOMA: a wearable device to assess the upper limb muscular activity in patients with neuromuscular disorders. Acta Myol.

[28] de Jesus MO, Ostolin TLVDP, Proença NL (2022). Self-administered six-minute walk test using a free smartphone app in asymptomatic adults: reliability and reproducibility. Int J Environ Res Public Health.

[29] Oldroyd AGS, Krogh NS, Dixon WG, Chinoy H (2022). Investigating characteristics of idiopathic inflammatory myopathy flares using daily symptom data collected via a smartphone app. Rheumatology (Oxford).

[30] Yoshida A, Kim M, Kuwana M, et al. Impaired physical function in patients with idiopathic inflammatory myopathies: results from the multicentre COVAD patient-reported e-survey. Rheumatology (Oxford). 2022:keac441. 10.1093/rheumatology/keac441 10.1093/rheumatology/keac441 PMC9384667 35920795

[31] Shinjo SK, Kim M, Hoff LS (2023). Pain in individuals with idiopathic inflammatory myopathies, other systemic autoimmune rheumatic diseases, and without rheumatic diseases: A report from the COVAD study. Int J Rheum Dis.

[32] Taubmann J, Knitza J, Müller F, et al. Rescue therapy of antisynthetase syndrome with CD19-targeted CAR-T-cells after failure of several B cell depleting antibodies. Rheumatology (Oxford). 2023:kead330. 10.1093/rheumatology/kead33010.1093/rheumatology/kead33037432378

[33] Müller F, Boeltz S, Knitza J (2023). CD19-targeted CAR T cells in refractory antisynthetase syndrome. Lancet.

[34] Nakajima T, Sankai Y, Takata S (2021). Cybernic treatment with wearable cyborg Hybrid Assistive Limb (HAL) improves ambulatory function in patients with slowly progressive rare neuromuscular diseases: a multicentre, randomised, controlled crossover trial for efficacy and safety (NCY-3001). Orphanet J Rare Dis.

[35] Just IA, Fries D, Loewe S (2023). Movement therapy in lung transplantation candidates assisted by a lightweight wearable robot. Assist Technol.

[36] Krusche M, Burmester GR, Knitza J (2020). Digital crowdsourcing: unleashing its power in rheumatology. Ann Rheum Dis.

[37] Joshi M, Naveen R, Jagtap K (2023). Assessment of quality and reliability of YouTube videos for patient and physician education on inflammatory myositis. Clin Rheumatol.

[38] Fedkov D, Berghofen A, Weiss C (2022). Efficacy and safety of a mobile app intervention in patients with inflammatory arthritis: a prospective pilot study. Rheumatol Int.

[39] Fazal ZZ, Sen P, Joshi M (2022). COVAD survey 2 long-term outcomes: unmet need and protocol. Rheumatol Int.

[40] Ziade N, Aoude M, Hmamouchi I, et al. Global disparities in the treatment of idiopathic inflammatory myopathies: results from an international online survey study. Rheumatology (Oxford). 2023:kead250. 10.1093/rheumatology/kead250 10.1093/rheumatology/kead250 37228012

[41] Hannah JR, Gordon PA, Galloway J (2022). Validation of methods to identify people with idiopathic inflammatory myopathies using hospital episode statistics. Rheumatol Adv Pract.

